# Infectious diseases epidemic threats and mass gatherings: refocusing global attention on the continuing spread of the Middle East Respiratory syndrome coronavirus (MERS-CoV)

**DOI:** 10.1186/s12916-016-0686-3

**Published:** 2016-09-07

**Authors:** Alimuddin Zumla, Abdulaziz N. Alagaili, Matthew Cotten, Esam I. Azhar

**Affiliations:** 1 Division of Infection and Immunity, University College London, London, UK; 2 NIHR Biomedical Research Centre, UCL Hospitals NHS Foundation Trust, London, UK; 3 KSU Mammals Research Chair, Zoology Department, College of Science, King Saud University, Riyadh, Saudi Arabia; 4 Viroscience Department, Erasmus Medical Center, Rotterdam, Netherlands; 5 Special Infectious Agents Unit, King Fahd Medical Research Centre, King Abdulaziz University, Jeddah, Saudi Arabia; 6 Medical Laboratory Technology Department, Faculty of Applied Medical Sciences, King Abdulaziz University, Jeddah, Saudi Arabia

**Keywords:** MERS-CoV, Mass gathering, Hajj, Infectious diseases, Epidemic transmission, Control

## Abstract

Media and World Health Organization (WHO) attention on Zika virus transmission at the 2016 Rio Olympic Games and the 2015 Ebola virus outbreak in West Africa diverted the attention of global public health authorities from other lethal infectious diseases with epidemic potential. Mass gatherings such as the annual Hajj pilgrimage hosted by Kingdom of Saudi Arabia attract huge crowds from all continents, creating high-risk conditions for the rapid global spread of infectious diseases. The highly lethal Middle Eastern respiratory syndrome coronavirus (MERS-CoV) remains in the WHO list of top emerging diseases likely to cause major epidemics. The 2015 MERS-CoV outbreak in South Korea, in which 184 MERS cases including 33 deaths occurred in 2 months, that was imported from the Middle East by a South Korean businessman was a wake-up call for the global community to refocus attention on MERS-CoV and other emerging and re-emerging infectious diseases with epidemic potential. The international donor community and Middle Eastern countries should make available resources for, and make a serious commitment to, taking forward a “One Health” global network for proactive surveillance, rapid detection, and prevention of MERS-CoV and other epidemic infectious diseases threats.

## Background

Mass gatherings at sporting [1, 2] and religious events [3] attract huge crowds, creating high-risk conditions for the rapid spread of infectious diseases. The 2000–2001 meningococcal meningitis outbreak after the Hajj pilgrimage [4] illustrated this threat of infectious diseases on global health security. The International Health Regulations Emergency Committee of the World Health Organization (WHO) declared Zika virus (ZKV) a Public Health Emergency of International Concern [5] on 1 February 2016. The media hype on ZKV transmission at the 2016 Rio Olympic Games diverted the attention of global public health authorities from other lethal infectious diseases with epidemic potential [6]. Attention and resources must now be refocused on the continuing epidemic threat of the highly lethal Middle East respiratory syndrome coronavirus (MERS-CoV) [7, 8].

## The global spread of MERS-CoV

Between 8 and 14 September 2016, the cities of Jeddah, Makkah, and Madinah, Kingdom of Saudi Arabia (KSA), hosted the annual Hajj pilgrimage [3]. An estimated 2 million pilgrims attended from 184 countries, lived in crowded conditions, and performed religious rites in close proximity, exposing themselves and the local Saudi population to a range of infectious diseases. MERS-CoV was first isolated and identified in a 68-year-old patient who died of pneumonia and multi-organ failure in Jeddah, KSA, in June 2012 [9]. This is the second time (after severe acute respiratory syndrome coronavirus, SARS-CoV) [10] in the 21st century that a coronavirus has emerged as a new lethal zoonotic pathogen of humans.

Serological studies show that camels have been infected with MERS-CoV for 20 years. MERS-CoV is a common infection in dromedary camels, and there is accumulating evidence that the sporadic human outbreaks are seeded by zoonotic infection from camels [11]. There have been intermittent MERS-CoV community cases [12] and hospital outbreaks [13–16], but no sustained epidemic [7]. Hospital case clusters of MERS-CoV represent the primary location where rapid human-to-human transmission of MERS-CoV have occurred; although limited spread among family members has been observed [17, 18]. SARS-CoV [10] was also predominantly spread through nosocomial transmission, but the epidemiological features of MERS-CoV remain less clear [19]. While 90 % of reported MERS-CoV cases have been from KSA, MERS-CoV has spread to 27 countries in Europe, North Africa, Asia, USA, and the Middle East. All cases had travel links with KSA or other countries in the Arabian Peninsula. As of 26 August 2016, 1,800 confirmed MERS-CoV cases have been reported to WHO from over 21 countries, including 640 deaths (35 % case fatality rate). MERS-CoV-related mortality is significantly increased in patients with comorbidities such as diabetes, renal disease, cardiac disease, lung and liver disease, or other immunosuppressive conditions [7, 20]. There are no known effective treatments or preventive vaccines for MERS-CoV [21].

## The continuing spread and epidemic threat of MERS-CoV

The risk of sustained person-to-person transmission appears to be very low and MERS-CoV is considered to have a low epidemic potential [20, 22]. Nevertheless, we have constant reminders of the epidemic threat of MERS-CoV from several hospital-associated outbreaks started by a single case [13–16, 23]. Of particular concern was the 2015 MERS-CoV outbreak in the Republic of South Korea [23, 24], in which 184 MERS cases (including 33 deaths) occurred in 2 months [23]. These were linked to a South Korean businessman who had travelled to four countries in the Middle East and contracted MERS-CoV infection. Falling ill after his return to Seoul, the patient waited in overcrowded hospital emergency rooms [24] and was responsible for MERS-CoV super-spreading events at five hospitals [25]. While this major outbreak should have been a wake-up call [26–28], the attention of global public health authorities at the time was diverted by the Ebola virus disease epidemic in West Africa [29] and, more recently, by the ZKV epidemic [30].

It remains an enigma that, while SARS-CoV spread rapidly globally and caused >8,000 cases and 775 deaths (10 % mortality) before disappearing within 8 months of discovery [10], MERS-CoV continues to circulate 4 years after first identification [9], causing intermittent community and hospital-associated outbreaks [19]. The exact mode of transmission to humans from camels and other possible animal sources remains undefined. Worryingly, the very high mortality and absence of specific MERS-CoV treatments or vaccines will seriously impact healthcare services of countries from which Hajj pilgrims originate if a Korea-like outbreak [23–25] occurs from returning pilgrims.

## MERS-CoV: vigilance, early detection, and infection control measures

The past three annual Hajj pilgrimages (14 October 2013, 3 October 2014, and 23 September 2015) have passed by without any increase in travel-related MERS-CoV cases [19]. While KSA authorities are on full alert for MERS-CoV, with their healthcare systems in place and resources to tackle MERS-CoV outbreaks, the healthcare institutions in the home countries of pilgrims need to remain vigilant for MERS-CoV cases [28]. Advice has been made available for pilgrims to take precautions to minimize health risks [31]. Pilgrims with chronic medical conditions such as diabetes or chronic heart, liver, and lung conditions, and those taking steroids or other immunosuppressive treatments are more susceptible to serious MERS-CoV disease and should seek medical advice before embarking on pilgrimage. The risk of MERS-CoV infection after exposure to camels or camel products is real [7, 11, 14]. Pilgrims must avoid visiting places where camels are found – highways between cities, camel farms and camel races, farms, barns, or market areas in any part of KSA. They should not consume raw camel milk or camel products or food that may be contaminated with animal secretions. In light of growing evidence, KSA authorities banned the slaughtering of camels for sacrifice and the movement of camels around Makkah and Madinah during the 2016 Hajj [32].

The risk of returning pilgrims infected with MERS-CoV to their home countries remains. Thus, health professionals and healthcare systems should remain vigilant at all times [28], and especially in the months after the end of the Hajj. Many pilgrims remain in KSA for several weeks after the Hajj, but because symptoms of MERS-CoV infection can occur up to 2 weeks after first infection [7], early detection is important. Pilgrims should be advised to seek medical attention promptly, and they should inform the doctor of their travel to KSA. As the Korean MERS-CoV outbreak showed [16], gaps in infection control measures are driving factors for nosocomial outbreaks. Early identification of the possibility of MERS-CoV and rapid implementation by healthcare workers of appropriate infection control measures for suspected cases is crucial to avoid outbreaks. Isolation of suspected and confirmed MERS-CoV cases managed under airborne-infection control precautions [33, 34] has been shown to be effective in containing nosocomial outbreaks [14–16, 24]. Contact tracing and screening should be undertaken by relevant public health authorities [33, 34].

## Need for more collaborative approach for optimal management, prevention, and control of MERS-CoV

MERS-CoV remains in the WHO list of top “emerging diseases likely to cause major epidemics” [35]. However, many questions on MERS-CoV epidemiology, pathogenesis, management, and control remain unanswered [36–38]. At long last, hope for filling the knowledge gaps and advancing research activities comes from the recent “MERS-CoV R&D Program” initiative, a joint endeavor by three KSA institutions: Ministry of Health, Ministry of Agriculture, and King Abdulaziz City for Science and Technology (KACST) [39]. This program has made competitive research grant funding available specifically to Saudi Arabian researchers to build local capacity. It is anticipated that this will lead to a more collaborative and coordinated international MERS-CoV response plan to better define MERS-CoV epidemiology, transmission dynamics, molecular evolution, pathogenesis, optimal treatment, and prevention interventions for humans and camels. Animal, human, and environmental factors play a crucial role in the persistence, continuing outbreaks, and evolution of MERS-CoV. All 184 countries from which pilgrims originate should strengthen their public health systems, and the international donor community, including the wealthy Middle Eastern countries, should make available resources for a “One Health” framework for early detection and prevention of any future epidemics of MERS-CoV and other zoonotic infectious diseases [40]. Only then will the risk of recurring global zoonotic epidemics be reduced.
